# Mendel’s Law Demonstrated

**Published:** 1915-01

**Authors:** 


### Mendel's Law Demonstrated.

Eugene Fischer, in a recent review of his investigations of a hybrid race, has shown how remarkably the principles of Mendel's law have been verified. His research is in a comparatively new field of labor and was the investigation of the product of miscegenation between Boers and Hottentots. The inheritance of unite characters in the Mendelian ratio was strikingly near the average of the theoretical calculation. So were the laws of dominants and recessives confirmed.

The author concludes that the mechanism of heredity is the same when races of mankind are crossed as when breeds of live stock and varieties of plants are hybridized.

Speaking further on his observations, he states, in reference to fecundity in the hybrids, that it appears not to be in the least diminished and that they are fertile with either one of the parent races.

As to whether a new race can be formed through crossing, the doctor's conclusions are that "the first generation of hybrids are certainly not a new race but merely a patchwork of characteristics inherited from their parents. But in a later generation appearances are very different. As the unite characters are separately inherited, the chance for a given individual to inherit all or most of those of one of the parental races becomes steadily smaller. The characteristics of the parent races, therefore, are still present after any numbers of generations, but the resemblance of individuals to either parent race has become less (excluding, for the moment, all question about the influence of environment). When a hybrid population has once become established, it, therefore, remains constant with respect to the inheritable anthropological characters."

Of prepotency, he says: "A prepotency of either of the parent races, a stronger tendency of the hybrids to resemble either one of them, does not exist. On the average, the hybrids occupy an exactly intermediate position. One cannot talk safely of a prepotency."

From these observations, Fischer does not conclude that there is the formation of a new race, but that mankind, as it exists today, forms but a single biological species.

This study by so eminent an authority deserves more than passing interest, as it is the first real practical example of Mendelia inheritance in man. Human inheritance, as Professor Conklin points out in "Phenomena of Inheritance," November *Popular Science Monthly,* must always be less secure than in the study of the lower animals, for the reason that there are no "pure lines," and that experiments are out of the question. We rely principally upon observation and statistics. The offspring in man, compared with some animal forms, are so few that it is difficult to determine what all the hereditary possibilities of a family may be. This makes the method of inheritance of many human characters very uncertain.

So far, sixty human traits have been recorded by Davenport and Plate, on being inherited by Mendelian laws.

Dr. Conklin regards these hereditary principles of Mendel as being almost as important for biology as the atomic theory of Dalton is for chemistry. He goes on to state: "By means of these principles, particular dissociations and recombinations of characters can be made with almost the same certainty as particular dissociations and recombinations of atoms can be made in chemical reactions. By means of these principles the hereditary constitution of organisms can be analyzed and the real resemblances and differences of various organisms determined. By means of these principles the once mysterious and apparently capricious phenomena of prepotency, atavism and reversion find a satisfactory explanation."

Before the establishment of Mendel's principles, heredity was as Balzac said, "a maize in which science loses itself." Much still remains to be discovered about inheritance, but the principles of Mendel have served as an Ariadne's thread to guide science through this maize of apparent contradictions and exceptions in which it was formerly lost.

